# DCB dissolution of iron oxides in aeolian dust deposits controlled by particle size rather than mineral species

**DOI:** 10.1038/s41598-022-06734-2

**Published:** 2022-02-18

**Authors:** Qianqian Yang, Xusheng Li, Zhiyong Han, Xiaoyong Wang, Wancang Zhao, Shuangwen Yi, Huayu Lu

**Affiliations:** 1 School of Geography and Ocean Science, Nanjing University, Nanjing, 210023 China; 2 School of Geographical Sciences, Southwest University, Chongqing, 400715 China

**Keywords:** Climate sciences, Environmental sciences

## Abstract

Dithionite-citrate-bicarbonate (DCB) treatment is a classical method for removing iron oxides from soil. The DCB-induced dissolution effects on iron oxides are controversial. In this paper, samples from a typical loess-paleosol sequence in the Chinese Loess Plateau (CLP) and from other aeolian dust deposits in southern China were collected, and changes in the grain size composition and magnetic properties of the samples after DCB treatment were analyzed. The results show that the dissolution of iron oxides in loess-paleosol samples from the CLP is highly grain size dependent. In addition to completely dissolving nanometer-sized pedogenic iron oxides (< 0.2 μm), the standard DCB procedure can also dissolve submicron- and micron-sized aeolian iron oxides (0.2–6 μm). For these aeolian iron oxides, the submicron-sized (0.2–1 μm) iron oxides are sufficiently dissolved, and the solubility of the micron-sized (1–6 μm) iron oxides decreases with increasing particle size. The dissolution of > 6 μm aeolian iron oxides is negligible. DCB can neither separate pedogenic iron oxides from aeolian iron oxides nor selectively dissolve magnetite or maghemite. Although the total amount of dissolved iron oxides in the profiles from southern China is higher than that in the LC profile from northern China, the submicron- and micron-sized aeolian iron oxides in the latter are more easily dissolved.

## Introduction

Iron oxides, iron hydroxides and their hydrates (hereinafter collectively referred to as iron oxides, which include magnetite, maghemite, hematite, goethite, etc.) are mainly products of chemical weathering of iron-containing silicate minerals and are widely distributed in the environment at the Earth’s surface^1^. The formation, transformation and preservation of iron oxides are sensitive to climate and environmental changes^2–10^. Therefore, the mineral morphology and chemical characteristics of iron oxides are important indicators of changes in the climate and environment during weathering and pedogenesis processes^11–14^.

The separation and removal of iron oxides is of great significance for the chemical analysis of soil and clay. Dithionite-citrate-bicarbonate (DCB) treatment is the most classical and widely used chemical technique for dissolving iron oxides in soil or clay^15–17^. DCB treatment removes iron oxides in samples through the following reactions: reducing ferric iron with sodium dithionite, chelating the reduced iron with sodium citrate, and buffering the solution (to maintain a pH value of approximately 7.3) with sodium bicarbonate^15^.

Aeolian dust deposits consisting of loess-paleosol sequences are one of the most complete and continuous continental paleoclimate archives in the world and contain abundant paleoclimate information^18–20^. With the rise of the study of loess environmental magnetism^21–32^, magnetic susceptibility has become widely used as a proxy index for paleoclimatic changes^4,33–36^. The changes in magnetic susceptibility essentially reflect the formation and transformation of iron oxides in aeolian deposits during weathering and pedogenesis. It is generally believed that the formation of fine ferromagnetic particles in the process of pedogenesis leads to the enhancement of paleosol magnetism^23,24,32,37–39^. The combination of the DCB method and loess environmental magnetism provides a new approach to further understand the mechanism by which iron oxides are transformed and the paleoclimatic implications of magnetic susceptibility for the process of pedogenesis^27,30,37,40,41^.

To date, many achievements have been made by cross-research involving DCB treatment and loess magnetism, but some problems have yet to be solved. For example, the threshold grain size of DCB-soluble iron oxides is often cited as 1 μm based on a study on synthetic samples, which showed that the standard DCB procedure dissolves fine magnetite particles (ca. < 1 μm) but leaves larger particles (ca. > 1 μm) essentially intact^16^. However, they only measured four particle sizes of magnetite samples (i.e., 0.19 μm, 1 μm, 14 μm and 55 μm) ^16^. The dissolution effect of DCB on iron oxides between 1 and 14 μm is not clear. Fine et al.^38^ suggest that the magnetic susceptibility of loess/paleosol sequences is mainly caused by ferromagnetic minerals, including two components. One is the DCB-soluble component, which represents grains near the superparamagnetic/single domain (SP/SD) boundary; the other is the DCB-resistant component, which represents multidomain (MD) grains. However, the DCB dissolution effect on pseudosingle domain (PSD) grains between these two components is not clear. Currently, the dissolution effect of DCB on coarse iron oxides is controversial^16,29,37,42–47^. Regarding the selectivity of DCB dissolution of iron oxides with different origins and mineral species, one study shows that the DCB can remove maghemite produced by pedogenesis but has no effect on protogenic magnetite; therefore, it is proposed that the magnetic susceptibility signals in the loess-paleosol sequences in China are mainly derived from pedogenic maghemite^37^. However, this proposal has been challenged by subsequent studies^42,48,49^. Maher and Thompson^42^ believe that DCB dissolution is dependent on grain size and mineral composition and is unable to distinguish between submicron magnetite and maghemite. Liu et al.^48^ proposed that DCB treatment not only destroys ultrafine ferrimagnets (of pedogenic origin) but also removes a significant fraction of the protogenic detrital material (of aeolian origin). Vidic et al.^49^ suggest that the DCB technique underestimates the contribution of the lithogenic component and overestimates that of the pedogenic component to the magnetic susceptibility.

In summary, the dissolution effects of the DCB treatment on iron oxides with different particle sizes (< 1 μm or > 1 μm), different origins (pedogenic origin or eolian origin) and different mineral species (magnetite or maghemite) are still controversial, which hinders the full understanding of the formation/transformation mechanism of iron oxides during the weathering/pedogenic processes of the loess-paleosol sequences. In addition, there is also a lack of comparative studies on the DCB-mediated dissolution of aeolian dust deposits under different climatic conditions, such as in southern versus northern China. In the past, the application of DCB to aeolian dust deposits was mostly carried out in combination with lithomagnetic analysis. In this paper, aeolian dust deposits from northern and southern China were selected as the research objects. Their grain size composition and magnetic characteristics before and after DCB treatment are compared in detail. The dissolution efficiency of the DCB treatment on iron oxides in a typical loess-paleosol sequence from the Chinese Loess Plateau (CLP) and the differences in dissolution between northern and southern China are discussed. This study will allow us to better understand the mechanism underlying the dissolution of iron oxides by DCB and promote the application of the DCB technique in paleoclimatic research.

## Materials and methods

### Profiles and samples

**Figure 1:**
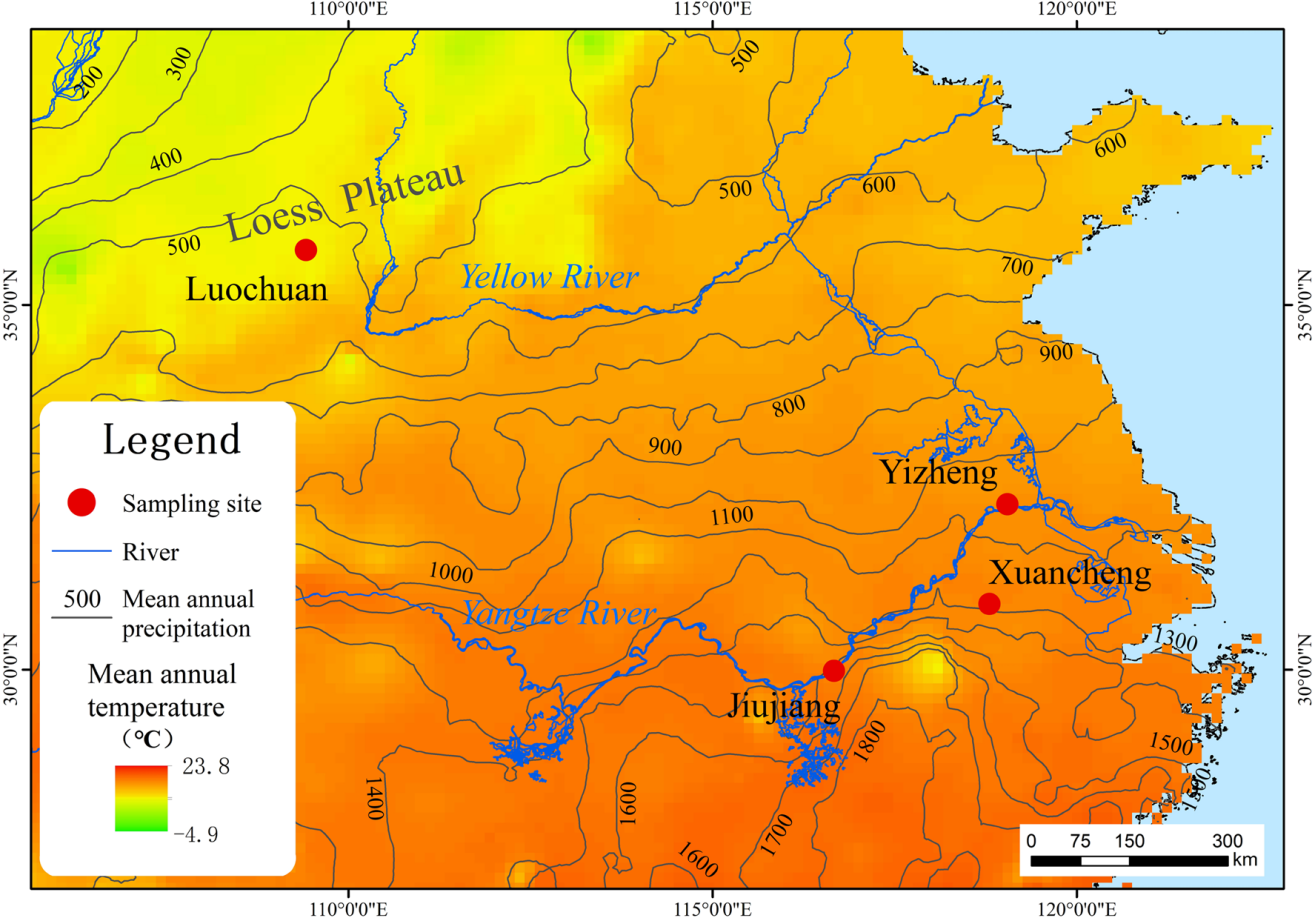
Locations of profile samplings. ArcGIS software (Version 10.1, www.esri.com/software/arcgis) was used to generate the figure. Base map source: 1:1 million national basic geographic database of China (https://www.webmap.cn/commres.do?method=result100W); Meteorological data source: China surface climate standard annual value data set (1981–2010) (http://data.cma.cn/data/cdcdetail/dataCode/SURF_CLI_CHN_MUL_MYER_19812010.html).

The aeolian dust samples were collected from four sites, namely, the Luochuan profile (LC, a famous loess-paleosol profile in the CLP)^50^; the Yizheng profile (YZ, a Xiashu loess profile in Jiangsu Province)^51^; the Jiujiang profile (JJ, another Xiashu loess profile in Jiangxi Province)^52^; and the Xuancheng profile (XC, a Pleistocene aeolian red earth profile in Anhui Province)^53^. The first site is in northern China, and the last three sites are in southern China. Information on the profiles and corresponding climate is shown in Fig. 1 and Table 1.

**Table 1:** Summary of aeolian dust deposit profiles and climate information.

Profile	Location	Longitude and latitude	Sample type	Climate	Mean annual temperature (°C)	Mean annual precipitation (mm)
	Chinese Loess Plateau					
Luochuan profile (LC)	Shaanxi Province	35°45' N, 109°25' E	Loess and paleosol	Warm temperate continental monsoon climate	10.0	592
	Southern China					
Yizheng profile (YZ)	Jiangsu Province	32°16' N, 119°03' E	Xiashu loess	Northern subtropical monsoon climate	15.7	1059
Jiujiang profile (JJ)	Jiangxi Province	29°59' N, 116°40' E	Xiashu loess	Central subtropical monsoon climate	17.1	1485
Xuancheng profile (XC)	Anhui Province	30°54' N, 118°48' E	Aeolian laterite	Central subtropical monsoon climate	16.1	1357

As shown in Table 2, fourteen loess-paleosol samples were collected from the LC profile (including 7 loess samples and 7 paleosol samples). Fifteen samples of the Xiashu loess were collected from the YZ profile. Nineteen samples were collected from the JJ profile. Nine samples of aeolian red earth were collected from the XC profile. Each of the 57 samples collected in this study weighed approximately 500 g. All the samples were air dried before further treatment.

**Table 2:** Sample collection information of four profiles.

Profiles	Sample No	Depth (m)	Strata/Lithology	Profiles	Sample No	Depth (m)	Strata/Lithology
Luochuan Profile (LC) n = 14	LC040	0.40	S0	Jiujiang Profile (JJ) n = 19	JJ005	0.05	L1
	LC085	0.85	L1LL1		JJ045	0.45	L1
	LC160	1.60	L1LL1		JJ205	2.05	L1
	LC395	3.95	L1SS1		JJ250	2.50	L1
	LC475	4.75	L1SS1		JJ355	3.55	S1
	LC824	8.24	S1		JJ525	5.25	L2
	LC1050	10.50	L2		JJ685	6.85	L2
	LC1130	11.30	L2		JJ760	7.60	L2
	LC1345	13.45	L2SS2		JJ900	9.00	S2SS1
	LC1620	16.20	S2SS2		JJ955	9.55	S2LL1
	LC1786	17.86	S2SS2		JJ990	9.90	S2SS2
	LC1930	19.30	L3		JJ1060	10.60	L3
	LC1995	19.95	L3		JJ1200	12.00	L3
	LC2015	20.15	L3		JJ1330	13.30	S3
Yizheng Profile (YZ) n = 15	YZ001	0.10	L1LL1		JJ1500	15.00	S3
	YZ005	0.50	L1LL1		JJ1610	16.10	L4
	YZ010	1.00	L1LL1		JJ1800	18.00	L4
	YZ015	1.50	L1SS1		JJ1990	19.90	L4
	YZ025	2.50	L1SS1		JJ2000	20.00	L4
	YZ036	3.60	L1SS1	Xuancheng Profile (XC) n = 9	XC-A005	1.00	Red earth Red earth Red earth
	YZ045	4.50	L1LL2		XC-A006	1.20	
	YZ055	5.50	L1LL2		XC-A009	1.80	
	YZ065	6.50	L1LL2		XC-A019	3.80	Vermicular red earth
	YZ070	7.00	S1		XC-A029	5.40	Vermicular red earth
	YZ085	8.50	S1		XC-C002	6.60	Vermicular red earth
	YZ100	10.00	L2		XC-C009	8.00	Vermicular red earth
	YZ108	10.80	S2		XC-C018	9.80	Vermicular red earth
	YZ110	11.00	L3		XC-C035	13.20	Vermicular red earth
	YZ120	12.00	S3				

### Methods

#### DCB treatment

An aliquot of each sample was treated with the DCB procedure^15^. (1) A suitable amount of the sample (0.2 g for each sample of the LC, YZ and JJ eolian loess profiles, 0.1 g for each sample of the XC eolian red earth profile) was placed into a beaker. A total of 40 ml of 0.3 mol/L sodium citrate and 5 ml of 1mol/L sodium bicarbonate were added to the beaker and dissolved in an ultrasonic cleaner at 80 ℃ in water bath. (2) A total of 1 g sodium dithionite was added, stirred and continuously oscillated for 16 min. (3) After the reaction, the suspension was then centrifuged at 2000 rpm for 15 min, and the samples were washed by centrifugation 3~4 times until the supernatant was clear and transparent. (4) The centrifuged sample was placed in a dryer at 40 ℃ and incubated for 24 hours.

#### Grain size measurement

Fifty-seven samples were tested for grain size. Two methods were used to pretreat the samples before the grain size measurement. One is a conventional technique without DCB treatment, and the resulting samples were called the pre-DCB samples. The other includes the DCB procedure, and the resulting samples were called the post-DCB samples. In general, the conventional pretreatment process includes the removal of organic matter and carbonate using H_2_O_2_ and HCl, respectively^54,55^. However, using HCl will partially dissolve iron oxides in the samples. In addition, HCl treatment is unnecessary and redundant for aeolian sediments from southern China because of their very low to nonexistent carbonate contents^56,57^. One later study suggested that pretreating postdepositionally modified aeolian sediments with HCl may result in misleading grain size distributions and should be avoided in standard analyses of loess-paleosol sequences^58^. Therefore, the pretreatment of LC, YZ, JJ and XC samples in this paper includes the application of only H_2_O_2_ instead of H_2_O_2_ + HCl.

After pretreatment, 10 ml of 0.05 mol/L (NaPO_3_)_6_ was used as the dispersant, and the samples were vibrated in an ultrasonic cleaner for 10 min before measurement using a Mastersizer 2000 laser particle size analyzer produced by Malvern Company, UK. The theoretical measurement range of this instrument is 0.02–2000 μm, and the actual measured data range is 0.2–1000 μm. The grain sizes were measured at the Laboratory of Land Surface Process of Nanjing University.

#### Rock magnetism measurement

Magnetic susceptibility (χ) of the samples before and after DCB treatment was measured with a Bartington MS2 meter at frequencies of 470 Hz (low frequency, χ_lf_) and 4700 Hz (high frequency, χ_hf_), respectively. Frequency-dependent susceptibility (χ_fd_) was defined as χ_fd_ = χ_lf_ − χ_hf_^32^. Unless otherwise indicated, χ refers to χ_lf_ in the following sections.

Of the 57 samples mentioned above, 8 representative samples, including one loess sample (LC085) and one paleosol sample (LC824) from the LC profile and six aeolian dust deposit samples from profiles YZ, JJ and XC, namely, YZ010, YZ070, JJ205, JJ1060, XC-A005 and XC-C009, were selected for hysteresis measurement before and after DCB treatment. Among these samples, LC085, LC824, YZ070, JJ205 and XC-A005 were selected for high-temperature magnetic susceptibility (χ-T curves) and measurement before and after DCB treatment. The hysteresis parameters were determined using a MicroMag 3900 Alternating Gradient Magnetometer (Princeton Measurements Corp., USA), with a measurement range of 50 μemu–10 emu and a sensitivity of 0.5 μemu. The magnetic field was cycled between ± 1.5 T. Saturation magnetization (Ms), saturation remanence (Mrs), coercivity (Bc) and coercivity of remanence (Bcr) of the samples were obtained after paramagnetic correction of hysteresis loops. High-temperature magnetic susceptibilities (χ-T) were measured using a KLY-3 s Kappabridge instrument equipped with a CS3 high-temperature furnace (Agico Ltd., Czech Republic) in an argon atmosphere. The maximum temperature was 700 °C, and the temperature increased at a rate of 4 °C/min. The rock magnetism measurements were performed at the Institute of Geology and Geophysics, Chinese Academy of Sciences.

#### Total iron and free iron measurement

Furthermore, 8 representative samples (2 from each profile) were selected to determine the contents of total iron (Fet) and free iron (Fed). Finely ground samples were digested in HF-HNO_3_-HClO_4_ to measure Fet, while Fed was extracted by DCB treatment. Iron in solution was determined with the *o*-phenanthroline method^59^. The Fet and Fed measurements were performed at the Institute of Soil Science, Chinese Academy of Sciences, and the experiments were carried out following the steps described in the reference^60^.

## Results

### Particle size changes pre- and post-DCB

#### Changes of grain size parameters

The results (Table 3) show that all samples become coarser after the DCB treatment and that the mean diameter and median diameter increase. After the DCB treatment, the mean diameter exhibits a larger variation range, increasing by an average of 22.90% in the LC profile. For the YZ, JJ and XC profiles from southern China, the mean diameter increases by 12.11%, 17.98% and 20.82%, respectively. The variation in the median diameter is relatively small. The average increases in the median diameter in the LC, YZ, JJ, and XC samples are 12.61%, 8.69%, 12.23% and 17.04%, respectively.

**Table 3:** Grain size distributions in aeolian dust deposits before DCB treatment (pre-DCB) and after DCB treatment (post-DCB).

Profiles and sediments	Items	0.2–1 μm (%)	1–2 μm (%)	2–3 μm (%)	3–4 μm (%)	< 4 μm (%)	4–8 μm (%)	8–16 μm (%)	16–32 μm (%)	> 32 μm (%)	Mean Diameter (μm)	Media diameter (μm)
LC profile	Pre-DCB	4.48 ± 0.57	4.93 ± 0.57	3.22 ± 0.40	4.29 ± 0.52	16.93 ± 2.05	9.83 ± 1.06	18.01 ± 1.19	28.67 ± 1.41	26.57 ± 3.48	14.09 ± 1.44	17.75 ± 2.02
Loess-paleosol (n = 14)	Post-DCB	2.03 ± 0.17	2.67 ± 0.23	2.01 ± 0.22	3.34 ± 0.40	10.05 ± 0.99	9.66 ± 1.13	19.79 ± 1.60	31.53 ± 0.90	28.97 ± 3.36	17.26 ± 1.36	19.92 ± 1.79
	Relative change/%	− 54.44 ± 2.84	− 45.68 ± 2.72	− 37.59 ± 3.47	− 21.93 ± 3.73	− 40.45 ± 2.63	− 1.75 ± 3.01	9.87 ± 4.38	10.14 ± 3.87	9.41 ± 5.81	22.90 ± 4.64	12.61 ± 4.65
YZ profile Xiashu loess (n = 15)	Pre-DCB	6.38 ± 0.55	6.41 ± 0.45	4.20 ± 0.18	5.56 ± 0.18	22.55 ± 1.21	12.26 ± 0.43	19.94 ± 0.83	26.81 ± 0.69	18.43 ± 1.33	10.57 ± 0.41	13.05 ± 0.53
	Post-DCB	4.24 ± 0.71	5.67 ± 0.72	3.95 ± 0.34	5.38 ± 0.25	19.23 ± 1.93	12.31 ± 0.43	20.73 ± 0.86	28.16 ± 0.68	19.57 ± 1.68	11.86 ± 0.76	14.19 ± 0.79
	Relative change/%	− 33.54 ± 9.11	− 11.64 ± 8.55	− 6.11 ± 6.33	− 3.31 ± 2.92	− 14.75 ± 6.82	0.41 ± 1.74	4.01 ± 3.44	5.04 ± 2.91	6.15 ± 3.59	12.11 ± 4.64	8.69 ± 2.91
JJ profile Xiashu loess (n = 19)	Pre-DCB	5.79 ± 0.80	6.26 ± 0.65	4.31 ± 0.29	5.85 ± 0.27	22.21 ± 1.86	12.64 ± 0.52	18.16 ± 0.82	24.65 ± 0.79	22.34 ± 1.91	11.30 ± 0.74	13.64 ± 1.04
	Post-DCB	2.89 ± 0.34	4.63 ± 0.36	3.81 ± 0.20	5.63 ± 0.24	16.97 ± 0.88	13.08 ± 0.58	19.69 ± 0.94	26.50 ± 0.55	23.77 ± 1.66	13.29 ± 0.55	15.32 ± 0.83
	Relative change/%	− 49.53 ± 6.23	− 25.68 ± 5.58	− 11.33 ± 5.09	− 3.67 ± 4.11	− 23.30 ± 5.03	3.47 ± 3.23	8.44 ± 2.28	7.64 ± 4.58	6.72 ± 6.14	17.98 ± 6.71	12.23 ± 8.33
XC profile Aeolian laterite (n = 9)	Pre-DCB	10.37 ± 1.46	9.97 ± 1.20	5.79 ± 0.68	6.88 ± 0.79	33.02 ± 3.86	14.08 ± 1.50	21.64 ± 2.00	20.27 ± 2.64	10.99 ± 8.17	7.92 ± 3.25	8.46 ± 2.28
	Post-DCB	6.32 ± 1.24	8.69 ± 1.23	5.52 ± 0.56	6.87 ± 0.66	27.40 ± 3.44	14.76 ± 1.50	23.55 ± 2.29	22.08 ± 2.61	12.21 ± 7.94	9.47 ± 3.54	9.79 ± 2.18
	Relative change/%	− 38.80 ± 10.90	− 12.61 ± 8.61	− 4.28 ± 5.06	0.04 ± 3.43	− 16.88 ± 6.11	5.00 ± 4.01	8.86 ± 4.28	9.22 ± 4.64	15.34 ± 9.44	20.82 ± 8.11	17.04 ± 7.64

#### Changes of different grain size fraction

**Figure 2:**
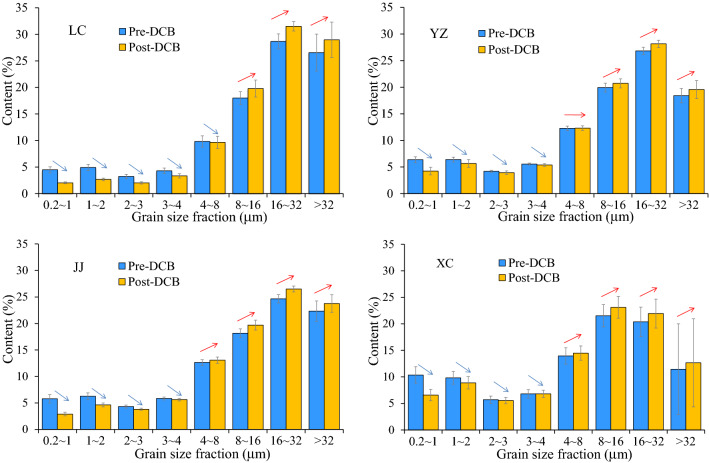
The contents of the different grain size fractions before and after DCB treatment.

The contents of fine fractions of < 4 μm (i.e., 0.2–1 μm, 1–2 μm, 2–3 μm and 3–4 μm) in the 4 profiles decrease after the DCB treatment, while those of coarse fractions of > 8 μm (i.e., 8–16 μm, 16–32 μm, and > 32 μm) increase (Table 3 and Fig. 2). The intermediate fraction (4–8 μm) shows a transitional variation between the fine and coarse fractions. However, the changes are inconsistent among the four profiles (e.g., the content of the 4–8 μm fraction decreases slightly in LC, increases slightly in JJ and XC, and remains almost unchanged in YZ).

**Figure 3:**
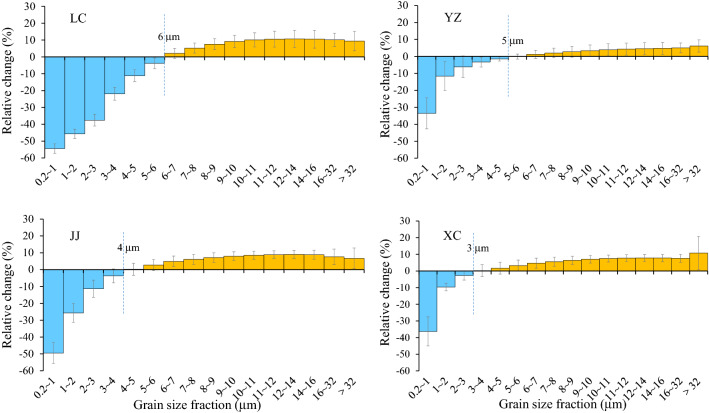
Relative changes in different grain size fractions after DCB treatment to those before DCB treatment.

**Figure 4:**
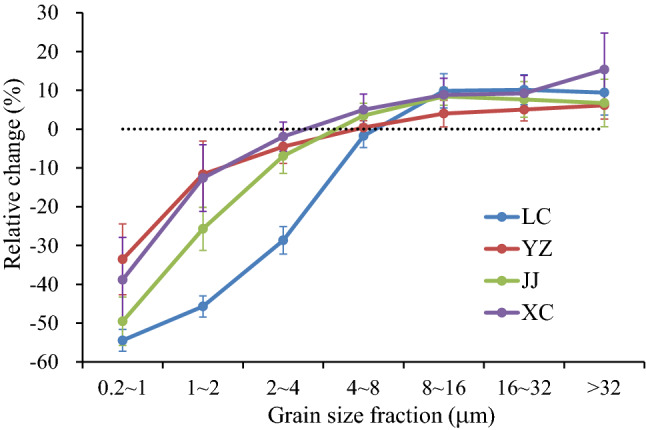
Relative changes in different grain size fractions from northern and southern China after DCB treatment compared to those before DCB treatment.

The relative changes in different grain size fractions after the DCB treatment are shown in Fig. 3. The relative change in the fine fractions in each profile is significantly larger than that in the coarse fractions. Generally, the finer the grain size is, the larger the relative change is. The relative changes in the < 4 μm fraction are − 14.75 to − 40.45%, and those in the 0.2–1 μm, 1–2 μm, 2–3 μm and 3–4 μm fractions are − 33.54 to − 54.44%, − 11.64 to − 45.68%, − 4.28 to − 37.59% and 0.04 to − 21.93%, respectively (Table 3). In the coarse fractions, the relative changes after the DCB treatment are much smaller, mostly less than 10%. The relative changes in the 8–16 μm, 16–32 μm and > 32 μm fractions are 4.01–9.87%, 5.04–10.14%, and 6.15–15.34%, respectively (Table 3). In the intermediate 4–8 μm fraction, the relative changes are − 1.75–5.00% (Table 3, Fig. 4).

#### Difference in grain size change between southern and northern China

Although the grain size of the aeolian deposits from both southern and northern China becomes coarser and the content of the fine fraction (< 4 μm) decreases after the DCB treatment, the content of the fine fraction in the samples from northern China decreases more than that in the samples from southern China (Fig. 3, Fig. 4). After the DCB treatment, the relative change in the < 4 μm component in LC was 40.45%, which was much higher than those in YZ (14.75%), JJ (23.30%) and XC (16.88%) (Table 3, Fig. 4). In addition, the particle size boundary between the decrease in fine particles and the increase in coarse particles from southern China (approximately 5 μm, 4 μm, and 3 μm in YZ, JJ and XC, respectively) corresponds to a slightly smaller grain size than that in the LC profile (approximately 6 μm) (Fig. 3).

Furthermore, there was no significant difference in the relative increase in the coarse fractions (i.e., 8–16 μm, 16–32 μm and > 32 μm) between southern and northern China after the DCB treatment, and there was no significant relationship between the relative change and the particle size (Fig. 3, Fig. 4).

### Changes in magnetism after DCB treatment

#### Magnetic susceptibility (χ) and its frequency dependence (χ_fd_)

After DCB treatment, the magnetic susceptibility (both χ_lf_ and χ_hf_) of all 8 representative samples from northern and southern China decreased significantly (Table 4). Although the χ value of pre-DCB samples varied greatly (36.63–232.38), the post-DCB samples exhibited a narrow range of χ values, all below 25. As with the post-DCB χ values, the post-DCB χ_fd_ values decrease greatly. The χ_fd_ value of pre-DCB samples varied from 1.04 to 37.83. However, after DCB treatment, the χ_fd_ values of all samples were close to 0 (all < 3). In terms of the average percentage decrease after DCB dissolution, the χ_lf_, χ_hf_ and χ_fd_ values decreased by 82.13%, 81.53% and 80.73%, respectively.

**Table 4:** Magnetic susceptibility and frequency dependence of the samples before and after DCB treatment﻿.

Sample	Pre-DCB (10^–8^ m^3^·kg^−1^)﻿			Post-DCB (10^–8^ m^3^·kg^−1^)			Percentage drop after DCB		
	χ_lf_	χ_hf_	χ_fd_	χ_lf_	χ_hf_	χ_fd_	χ_lf_ (%)	χ_hf_ (%)	χ_fd_ (%)
LC085	65.92	57.91	8.01	15.96	15.27	0.69	75.79	73.63	91.39
LC824	232.38	208.78	23.60	19.67	18.81	0.86	91.54	90.99	96.36
JJ205	39.31	36.64	2.68	7.78	7.21	0.57	80.21	80.32	78.73
JJ1060	45.27	44.24	1.04	18.70	17.79	0.91	58.69	59.79	12.50
YZ010	158.07	143.24	14.83	22.68	21.54	1.14	85.65	84.96	92.31
YZ070	206.00	168.18	37.83	20.51	20.51	0	90.04	87.80	100.00
XC-A005	149.75	131.65	18.10	14.28	11.46	2.82	90.46	91.30	84.42
XC-C009	36.63	29.92	6.71	5.61	4.95	0.66	84.68	83.46	90.16
Average							82.13	81.53	80.73

#### Temperature dependence of magnetic susceptibility (χ-T)

**Figure 5:**
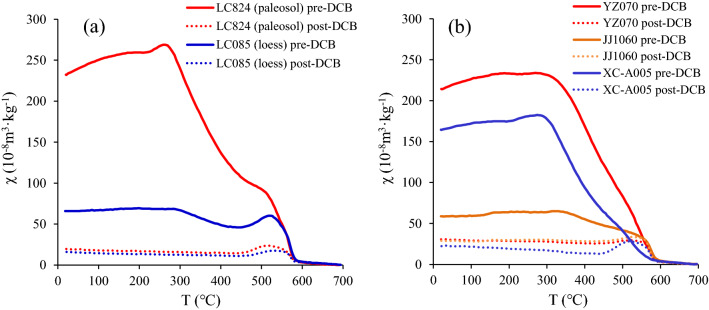
Heating curves of high-temperature magnetic susceptibility (χ-T) before and after DCB treatment. (a). LC profile in the CLP; (b). YZ, JJ, XC profiles from southern China.

Figure 5(a) shows the heating curves of the susceptibility in the LC profile. In samples without the DCB treatment, the χ values of the paleosol sample (LC824) decrease rapidly over the temperature range of 300–450 °C, and the loess sample (LC085) exhibits a slight decrease over the same interval. Therefore, a large amount of pedogenic maghemite is present in the paleosol, and only a small amount is present in the loess. The χ values of the paleosol and loess samples near the magnetite Curie point of 580 °C decrease sharply and form a significant inflection point, indicating that magnetite contributes significantly to the magnetic susceptibility. After the DCB treatment, the magnetic susceptibility is substantially lower, and the heating curves of loess and paleosol tend to coincide. The curves of the DCB-treated loess and paleosol samples are nearly horizontal over the temperature range of 300–450 °C, indicating that the maghemite has been fully dissolved by the DCB. There is still an obvious decline in the magnetic susceptibility near 580 °C, but the decline is less than that before the DCB treatment, indicating that some (fine) magnetite particles are dissolved by the DCB but that some magnetite particles still remain after the DCB treatment.

The χ-T curves of samples in three profiles of southern China also confirm the full dissolution of maghemite by DCB treatment (Fig. 5b), which is consistent with the LC profile of the CLP. However, the DCB-mediated dissolution of magnetite from southern China is obviously weaker than that of the LC profile from the CLP.

All samples treated with DCB have an obvious small peak at 450–550 °C. This phenomenon has also been reported in previous studies^27,29,47^. There are differences in the interpretation of this peak, which was called the Hopkinson peak by Deng et al.^29^. Sun et al.^27^ considered that DCB chemicals affect the structure of magnetite or remove the outermost stressed layers of magnetite grains, and then, magnetite is more susceptible to the Hopkinson effect. Lü et al.^47^ proposed that it is due to the formation of new magnetic minerals during the heating process. We believe that the appearance of this small peak is affected not only by the Hopkinson effect in single domain (SD) just below its Curie point^61,62^ but also by the production of magnetite or other higher susceptibility phases during heating^62^.

#### Hysteresis properties

**Figure 6:**
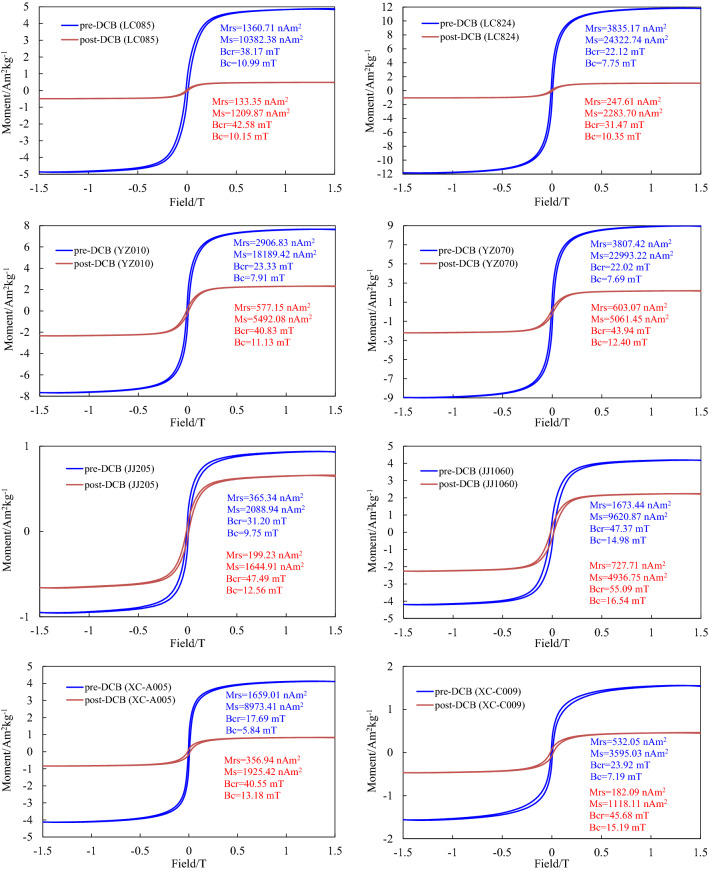
Hysteresis loops for representative samples before and after DCB treatment. Paramagnetic contributions were subtracted. The hysteresis parameters were measured up to ± 1.5 T.

Hysteresis loop analysis can provide important information about magnetic mineral types and domain states^64^. Before DCB treatment, the hysteresis loops of representative samples were close at 300–500 mT with slender (narrow) shapes (Fig. 6), indicating that the low-coercive ferrimagnetic components (magnetite and maghemite) were dominant in these samples (the XC-C009 sample was not close at 500 mT and showed a slight wasp-waist shape, suggesting a considerable amount of magnetic minerals with high coercivity). The hysteresis loops of all post-DCB samples were close at approximately 150–250 mT, indicating a predominant ferrimagnetic phase. However, compared with the pre-DCB samples, the slope of the hysteresis loop obviously changed from steep to slow, suggesting that the particle size of ferromagnetic minerals in the residue after DCB treatment became coarser. The values of hysteresis parameters Mrs and Ms decrease significantly for the post-DCB samples, while an increase in Bcr and Bc after DCB treatment may be due to the great dissolution of fine magnetic mineral particles (including ferromagnetic minerals and antiferromagnetic minerals).

**Figure 7:**
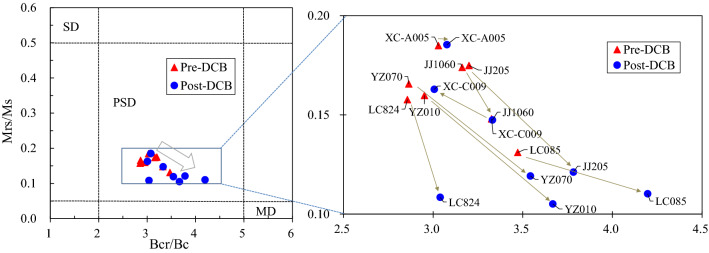
Hysteresis ratios plotted on a Day diagram of representative samples before and after DCB treatment. (The arrow in the left figure represents the trend of changes before and after DCB treatment, and the arrows in the right figure are the specific changes before and after DCB treatment for each sample).

The values of Mrs/Ms versus Bcr/Bc can be plotted on a Day diagram to analyze the mean particle size range of magnetic minerals. It was proposed by Day et al.^65^, and then Dunlop^66^ modified its parameters. In this paper, the improved Day diagram is used to analyze the magnetic domain state (Fig. 7). The Bcr/Bc ratio and Mrs/Ms ratio of typical samples from the four profiles ranged from 2.86 to 4.20 and 0.11 to 0.23. Before and after DCB treatment, all samples fall into the pseudosingle domain (PSD) region in the Day diagram, which indicates that the average domain state of magnetic minerals is a pseudosingle domain. However, after the DCB treatment, the sample points significantly shifted to the direction of coarser particles (MD), which confirmed that DCB treatment significantly coarsened the magnetic mineral particles.

### Total iron and free iron

The total iron content (Fet), free iron content (Fed) and Fed/Fet of 8 representative samples from four profiles are shown in Table 5. In general, the Fed and Fed/Fet of the three southern profiles are significantly higher than those in the northern LC profile. In terms of Fet, except for the MD205 and XC-A005 samples, which are slightly lower than LC824, the other samples are also higher in the southern profile than in the LC profile.

**Table 5:** Fed, Fet and Fed/Fet of representative samples in the four profiles.

Profile	Sample	Fed (g kg^−1^)	Fet (g kg^−1^)	Fed/Fet(%)
LC	LC085	7.40	33.57	22.04
	LC824	12.31	45.79	26.89
YZ	YZ010	14.86	48.36	30.72
	YZ070	18.40	51.74	35.57
JJ	JJ205	13.71	34.44	39.80
	JJ1060	19.52	48.81	39.99
XC	XC-A005	25.03	45.71	54.75
	XC-C009	35.18	58.54	60.10

## Discussion

### Grain size effect on iron oxide dissolution by DCB

The change in the grain size of aeolian dust deposits after the DCB treatment is clearly due to the dissolution of iron oxides. There are two possible dissolution mechanisms. The first is that the finest iron oxide particles are completely dissolved by DCB, and the second is that the coarse-grained iron oxide particles are partly dissolved by DCB, e.g., the outer layer of the particles is dissolved^47^. Both dissolution mechanisms led to a decrease in the content of the corresponding grain size fractions. The grain size distribution of all DCB-treated samples exhibits a coarsening trend, which can be explained by the disappearance of the fine-graded component through dissolution and an increase in the coarse-grained component. The decrease in the fine-grained component is certainly caused by the complete or partial dissolution of fine iron oxides. However, the increase in the coarse-grained component cannot be true because no mechanism can explain the enlargement of particles during the DCB treatment. Thus, the relative increase in the coarse-grained component is merely due to the decrease in the fine-grained component. Therefore, we can obtain direct evidence of the dissolution effect of DCB by analyzing the variation of the fine-grained component after the DCB treatment. Correspondingly, changes in magnetic properties (such as hysteresis loops) of samples after DCB treatment also provide indirect evidence of residue coarsening, indicating that fine magnetic mineral particles are dissolved or/and coarse iron oxide particles are partially dissolved.

Unlike previous studies^16^, the results show that the dissolution of iron oxides can take place for particles larger than 1 μm by DCB treatment. Taking the loess and paleosol samples from the typical LC profile from northern China as an example, the DCB treatment can dissolve iron oxide particles that are < 6 μm in size. The content of < 1 μm (i.e., 0.2–1 μm) decreases significantly, and its relative change exceeds 50%, suggesting that DCB can sufficiently dissolve submicron-sized iron oxides. The relative change in each fraction between 1 μm and 6 μm (i.e., 1–2 μm, 2–3 μm, 3–4 μm, 4–5 μm, and 5–6 μm) after the DCB treatment decreases with increasing grain size, indicating that micron-sized iron oxides are partly dissolved and that the dissolution efficiency decreases with increasing particle size. The main reason may be that the dissolution efficiency decreases with decreasing specific surface area (i.e., mass surface area) as the iron oxide grain size increases; thus, for coarse grains, only the outer layer can be dissolved by the DCB treatment^43,47^. For coarse-grained particles with sizes greater than 6 μm, dissolution by DCB is negligible.

### Can DCB separate pedogenic iron oxides from aeolian iron oxides and selectively dissolve maghemite or magnetite?

**Figure 8:**
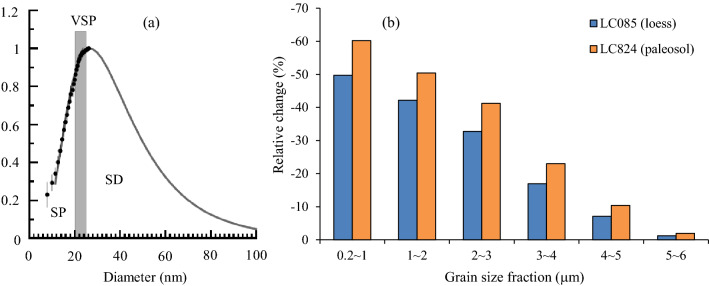
(**a**) Grain size distribution of pedogenic particles in the Chinese loess. The thick shaded bar represents a range of grain sizes for viscous superparamagnetic (VSP) particles^67^. (**b**) Relative change in each grain size fraction induced by DCB for the typical loess and paleosol samples.

There are two origins of iron oxides in aeolian dust deposits, i.e., pedogenic origin and aeolian (lithogenic) origin, and the pedogenic component primarily records the paleoclimatic signals^49^. The grain size distribution of pedogenic iron oxides in paleosols is stable, and these iron oxides are mainly superparamagnetic domain (SP) and single domain (SD) ferrimagnetic mineral particles, which are the main contributors to magnetic susceptibility^23,24,38,67^. The dominant grain size maximum is estimated to be just above the SP/SD threshold (~ 20–25 nm) but can extend to the upper boundary of the SD particles at 100 nm^67^ (Fig. 8a). In addition, Liu et al.^68^ noted that the lower grain size limit of aeolian magnetic particles is approximately 100–300 nm. Although the laser particle size analyzer cannot detect nanosized particles below 0.2 μm, the DCB dissolution of iron oxides of < 0.2 μm can be inferred from the change in magnetic susceptibility (χ). In two representative samples – LC824 (paleosol) and LC085 (loess) – from the LC profile, the magnetic susceptibility (χ) values before the DCB treatment are 232.4 × 10^–8^ m^3^·kg^−1^ (maximum in the samples of LC profile) and 65.9 × 10^–8^ m^3^·kg^−1^ (minimum in the samples of L1 stratum of LC profile), respectively. The χ value of the paleosol sample is 253% higher than that of the loess sample, and this difference is mainly due to the formation of ferrimagnetic minerals during pedogenesis. After the DCB treatment, the magnetic susceptibility of these two samples decreases to similar values (19.7 × 10^–8^ m^3^·kg^−1^ and 16.0 × 10^–8^ m^3^·kg^−1^), representing decreases of 212.7 × 10^–8^ m^3^/kg and 49.9 × 10^–8^ m^3^/kg, respectively.

However, the grain size measurements show that there was no particularly significant difference in the relative content changes for 0.2–6 μm particles between the loess and the paleosol after the DCB treatment (Fig. 8b), suggesting that the 0.2–6 μm iron oxides that were dissolved by DCB are not pedogenic in origin. The pedogenic iron oxides are therefore mainly extremely fine-grained particles less than 0.2 μm in size and were thoroughly dissolved by the DCB treatment. This dissolution is reflected by the changes in the magnetic susceptibility (significant decrease in χ after the DCB treatment) rather than by changes in the grain size.

**Figure 9:**
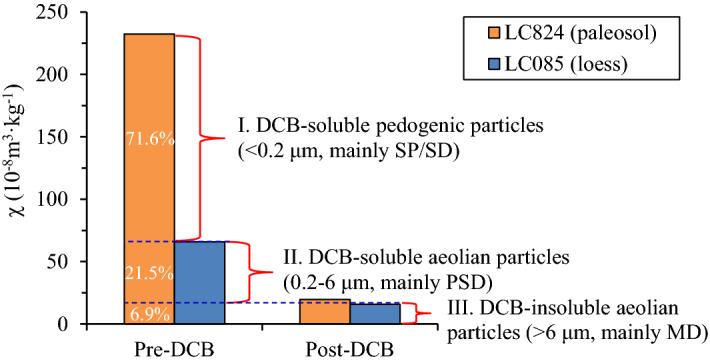
Three iron oxide components in the loess-paleosol LC profile classified according to the response of the magnetic susceptibility to DCB dissolution.

Therefore, we can divide the iron oxides in paleosols into three components according to the response of the magnetic susceptibility to DCB dissolution (Fig. 9). Component (I) is pedogenic iron oxides, mainly SP and SD ferrimagnetic mineral particles less than 0.2 μm, which can be completely dissolved by DCB; component (II) is DCB-soluble aeolian iron oxides, mainly PSD ferrimagnetic mineral particles of 0.2–6 μm; and component (III) is DCB-insoluble aeolian iron oxides, mainly coarse-grained particles of > 6 μm ferrimagnetic minerals (mainly multidomain, MD). If loess is regarded as aeolian deposits that are nearly unaffected by pedogenic processes (in actuality, the studied loess has also undergone weak pedogenesis), then component (I) enhances the magnetic susceptibility signal in the paleosol sample compared to the loess sample, accounting for approximately 71.6% of the χ values in the paleosol based on LC824 and LC085 as examples. The decrease in magnetic susceptibility of the loess sample after DCB treatment is attributed to the dissolution of component (II), accounting for approximately 21.5% of the χ values of the paleosol. The remaining magnetic susceptibility of the paleosol and loess samples after DCB treatment, accounting for approximately 6.9% of the χ values of the paleosol, is contributed by component (III) (Fig. 9). The above analysis shows that DCB retreatment cannot completely separate iron oxides of pedogenic and aeolian origins. In addition to nanosized SP and SD pedogenic particles (< 0.2 μm), the standard DCB procedure can also dissolve aeolian dust iron oxides in the form of submicron- and micron-sized PSD particles (0.2–6 μm). Vidic et al.^49^ also found that the DCB method overestimates the pedogenic component and underestimates the lithogenic component of magnetic susceptibility. We conclude that the existence of component (II) is the cause of the overestimation of the pedogenic component. Considering that the loess has also actually undergone weak pedogenesis, the contribution of component (II) to the magnetic susceptibility signal should be slightly lower than that estimated above.

The experimental results of the χ-T curve show that DCB treatment can fully dissolve maghemite grains, but the dissolution of magnetite is relatively more complicated. Parameter χ_fd_ is preferentially sensitive to a fairly narrow grain size range near the SP/SD (~ 20–25 nm for magnetite) boundary^30,69^. It can therefore be used to estimate whether SP material is present in a sample^70^, although it provides only a minimum estimate. The χ_fd_ values of all DCB-treated samples are close to 0, indicating that there is no superfine magnetite in the post-DCB samples. However, χ-T curve results confirmed that some magnetite grains remained in the residue after the DCB treatment. In combination with the change in grain size, we believe that the fine-grained magnetite was dissolved preferentially, while the coarse-grained magnetite was not fully dissolved. Therefore, DCB can dissolve both maghemite and magnetite in aeolian dust deposits in the CLP. Although maghemite seems to be easier to dissolve than magnetite, DCB does not selectively dissolve particular iron oxide minerals. The difference in dissolution efficiency between maghemite and magnetite should be attributed to their different particle size distributions. This is consistent with the view that paleosols contain more fine-grained pedogenic maghemite.

Hu et al.^71^ revealed that nanosized pedogenic hematite is the first mineral to be dissolved by DCB and that the dissolution of maghemite and magnetite was in the order of SP-SD-PSD. This particle size-dependent dissolution behavior forms a dissolution path on the Day diagram. Therefore, the so-called selective dissolution of iron oxides by DCB may essentially reflect the control of particle size on the dissolution efficiency of DCB. Iron oxide particles with small diameters, large specific surface areas and low crystallinity more easily react with solvents and can be dissolved preferentially. Therefore, Van Oorschot and Dekkers^44^ once proposed that the DCB procedure is sometimes more suitable for distinguishing grain size than for distinguishing between minerals.

Similar to the findings of this paper, recent studies have shown that chemical sequential extraction methods cannot accurately dissolve the targeted Fe minerals in natural sediments and sedimentary rocks. Other factors, such as particle size (as shown in this study), degree of crystallinity, and the presence or absence of organic matter, greatly affect differences in solubility between natural and synthetic forms of the same mineral, between different natural samples of the same mineral, and even between identical samples^72–74^. Our results once again highlight the subtlety and complexity of dealing with natural samples that contain diverse mineral assemblages^74^. As pointed out by Hepburn et al.^72^, the behavior of Fe minerals in the extraction method is clearly more dependent on the properties of the mineral than the specific mineral itself. These results remind us that it is necessary to use other independent methods (such as magnetic methods) to evaluate mineralogy following chemical extraction.

### Difference in DCB dissolution between southern and northern China

**Figure 10:**
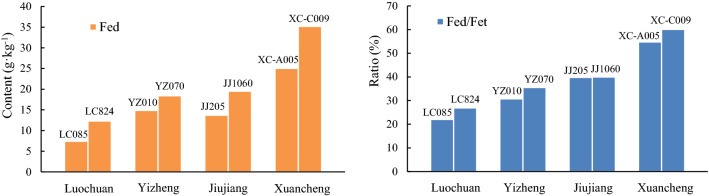
Comparison of Fed and Fed/Fet of representative samples from different profiles.

The grain size changes in aeolian dust deposits in southern China due to the DCB treatment are generally similar to those in the LC profile. There are also some differences between southern and northern China. The upper limit of grain size for the DCB-mediated dissolution of iron oxides in LC samples (6 μm) is slightly higher than that in YZ, JJ and XC samples from southern China (5 μm, 4 μm, and 3 μm, respectively) (Fig. 3). In addition, after the DCB treatment, the decrease in the fine-grained component from northern China was larger than that from southern China. However, the DCB-extractable Fe (Fed) and the ratio between DCB-extractable Fe and total Fe (Fed/Fet) are clearly higher in the YZ, JJ and XC samples than in the LC samples (Fig. 10), reflecting the higher intensity of weathering and pedogenesis in these profiles of southern China and the proportionately greater dissolution of iron oxides by the DCB treatment. These results reveal that although the total dissolved amount of iron oxides in these profiles of southern China is higher, the submicron- and micron-sized iron oxides in the LC profile of northern China seem to be more easily dissolved by DCB. Consistent with this, the χ-T curve of aeolian dust deposits (Fig. 5b) also shows that the dissolution of magnetite in samples from these profiles of southern China is weaker than those from the LC profile of the CLP.

These differences may be attributed to the difference in the occurrence state of submicron- to micron-sized iron oxides. In the north, the dispersed state may be dominant, while in the south, the cemented state may be dominant. Iron oxides can influence soil structure through cementation, and the cementation process mainly appears in acidic soil environments^75^. Under the humid and hot climate conditions in the south, stronger pedogenesis produces more nanosized iron oxides (such as hematite), which have a strong ability to cement soil particles and lead to the formation of very stable aggregates by cementing together clay minerals, such as kaolinite and montmorillonite. These aggregates are difficult to disperse even when using NaOH and an ultrasonic cleaning machine^76^. In addition, the amount of iron coating in the soil increases with increasing Fed, which makes the soil structure tend to be compact and gradually reduces the soil porosity. These effects may be disadvantageous to DCB dissolution.

Ferrimagnetic minerals (magnetite and maghemite) are the main contributors to the magnetic susceptibility signal; therefore, we mainly discuss the DCB dissolution of these two minerals in this paper. Compared to ferrimagnetic minerals, antiferromagnetic minerals (such as hematite and goethite) contribute less to magnetic susceptibility, but their contents in soil may be higher than those of ferrimagnetic minerals, especially in southern China. For the DCB dissolution effect of antiferromagnetic minerals, we have previously performed relevant research based on the method of diffuse reflectance spectroscopy^52^. The results show that DCB treatment can fully remove both hematite and goethite from the loess-paleosol samples from the CLP of northern China. For the southern loess samples, DCB treatment can only completely remove hematite but cannot fully dissolve the goethite in the samples. In addition to the abovementioned cause of the difference in the occurrence state, this may also be related to the presence of some goethite with better crystallinity and coarser grains in the southern loess-paleosol samples^52^.

## Conclusions

Our study provides direct evidence of the dissolution effect of DCB treatment on the iron oxides of aeolian dust deposits in China. The particle size and magnetism analysis show that the DCB dissolution of iron oxides in a loess-paleosol sequence in the CLP is significantly dependent on the grain size. In addition to nanosized pedogenic iron oxides (< 0.2 μm), aeolian iron oxides of submicron and micron size (0.2–6 μm) can also be dissolved by standard DCB treatment. Among these iron oxide particles, submicron-sized (0.2–1 μm) iron oxides are sufficiently dissolved, and micron-sized (1–6 μm) iron oxides are partly dissolved (the outer layer of the particle is dissolved) with a decrease in dissolution with increasing grain size. The dissolution of > 6 μm aeolian iron oxides is negligible. Therefore, DCB can neither completely separate pedogenic and aeolian iron oxides nor selectively dissolve magnetite or maghemite, and its dissolution efficiency is mainly dependent on the grain size of the iron oxides. These results remind us that it is necessary to use other independent methods (such as magnetic methods) to evaluate mineralogy following chemical extraction. In addition, the DCB dissolution of aeolian dust deposits reveals some differences between southern and northern China. The submicron- and micron-sized aeolian iron oxides from northern China seem to be more easily dissolved by DCB than those from southern China. This difference may be due to the difference in the presence of submicron- to micron-sized iron oxides, which are mainly present in a dispersed state in the CLP and in a partly cemented state in southern China.
